# Effect of glucagon-like peptide-1 receptor agonist on insulin secretion index and serum Wnt5a protein in patients with new-onset type 2 diabetes mellitus

**DOI:** 10.1007/s40200-022-01175-0

**Published:** 2023-01-03

**Authors:** Wei Xu, Y. Q. Sang, X. K. Liu, H. F. Geng, Ben Wang, Li Shi, Q. Q. Qiu, T. P. Yu, Yan Zhang, Xia Zhang, Lin Li, Qing Li, Jun Liang

**Affiliations:** 1 Department of Endocrinology, Xuzhou Central Hospital, Xuzhou Institute of Medical Sciences, Xuzhou Clinical School of Nanjing Medical University, Affiliated Hospital of Medical School of Southeast University, Jiefang Road 199#, Xuzhou, Jiangsu China; 2 Xuzhou Medical University, Xuzhou, China; 3 Bengbu Medical College, Bengbu, China

**Keywords:** Type 2 diabetes mellitus, Wingless-type MMTV integration site family member 5a, Glucagon-like peptide-1 receptor agonists, Homeostasis model assessment- β

## Abstract

**Objective:**

Previous studies have found that wnt5a promotes β-cell insulin secretion and reduced concentrations in patients with type 2 diabetes. GLP-1RA (Glucagon-like peptide-1 receptor agonists) can regulate insulin secretion. However, the evidence that GLP-1RA affect insulin secretion through the Wnt5a is inconclusive. Therefore, this study aimed to evaluate the effect of GLP-1 RA on wnt5a levels in patients with type 2 diabetes.

**Methods:**

A total of 56 onset diabetics were selected our study, 29 of them were treated by GLP-1RAs (1.2mg subcutaneous injection once a day, liraglutide, Novo Nordisk), the rest (27 case) treated by Metformin (0.5 g twice a day, Glucophage, Merck). Individuals who were using medications to manage platelet (Aspirin) and cholesterol (Statins) were enrolled and continued treatment throughout the study.

**Results:**

Our study found that the waist circumference and insulin secretion index in the GLP-1RA intervention group were significantly increased, and the insulin resistance index was lower than that of the control group. More interestingly, the serum Wnt5a protein level increased dramatically after the GLP-1RA intervention, and the level of Secreted frizzled-related protein 5 (Sfrp5) decreased compared with the control group. Multivariate linear regression analysis showed that the change of HOMA-β (Homeostasis model assessment- β) was significantly correlated with the changes of Wnt5a and Sfrp5, and the change of Wnt5a protein was positively correlated with HOMA-β.

**Conclusion:**

Our results confirmed that GLP-1RA may improve HOMA-β in patients with type 2 diabetes by affecting the level of Wnt5a protein.

## Introduction

Type 2 diabetes has become a severe public health problem globally, and the complications of diabetes have brought a severe burden to patients [1]. Insulin resistance and / or insufficient insulin secretion are its pathogenesis [2]. Studies have confirmed that glucose toxicity, lipotoxicity, low-level inflammation, and oxidative stress are all involved in the pathological mechanism of pancreatic beta-cell secretion disorders [3–6]. However, the specific mechanism of insulin secretion regulation is still a hot spot pursued by diabetes researchers worldwide. In recent years, the relationship between glucagon-like peptide-1 and insulin secretion has gradually been recognized by researchers.

The function of pancreatic islets is simply the ability of pancreatic beta cells to secrete insulin. Multiple hormones and proteins (Insulin, glucagon, growth hormone, somatostatin, adiponectin, etc) in the bodywork together to maintain blood glucose at a relatively stable level. In clinical studies, serum insulin and C-peptide levels are usually tested to assess islet function [7]. There are many methods for evaluating pancreatic islet function currently used in clinical practice, such as the positive glucose clamp test [8], the mini-model procedure [9, 10], the area under the curve (AUC) following an oral glucose tolerance test (OGTT) [11], and the arginine test [12]. However, due to the complexity of the above methods for detection, Homeostatic model assessment (HOMA) is usually used to explain the islet function of patients in clinical practice. In the study published by Turner et al. in 1985 in Diabetologia, they used the method and formula to calculate the HOMA index by measuring the individual’s fasting blood glucose level and fasting insulin level. HOMA is divided into three categories: HOMA-IR, HOMA- IS and HOMA-β. HOMA-β is an index used to evaluate the function of an individual’s islet β-cells and is currently a commonly used formula for assessing insulin secretion in clinical practice [13].

Wnt Family Member 5A (Wnt5a) is one of the crucial members of the non-classical Wnt family and participates in many physiological and pathological processes, such as tissue fibrosis, tumours and other diseases [14–17]. As an important cytokine secreted by adipose tissue, Wnt5a has attracted the attention of researchers in research related to diabetes. Our previous studies have confirmed that the level of Wnt5a is significantly reduced in patients with newly-onset type 2 diabetes [18]. Wnt5a can regulate the insulin secretion of the beta-cell through the non-classical Wnt/Ca++ pathway and Fox01 signalling pathway [19, 20]. Metformin can increase the level of serum Wnt5a protein in patients with type 2 diabetes in addition to improving blood glucose (Unpublished). Although our team has a preliminary understanding of the mechanism of insulin secretion in Wnt5a and beta cells, the effect of GLP-1RA on Wnt5a protein in diabetic patients is still unclear.

GLP-1 (glucagon-like peptide-1), a brain-gut peptide secreted by ileal endocrine cells, can inhibit gastric emptying and reduce bowel movement, so it helps control food intake and reduce weight [21]. Previous studies have confirmed GLP -1 promotes insulin gene expression and biosynthesis via the Pancreatic and Duodenal Homeobox 1(PDX1) signaling pathway [22, 23]. GLP-1 attenuates endoplasmic reticulum (ER) stress by activating PKA and affects β cell function and survival [24]. GLP-1 acts as a growth factor by promoting β cell proliferation and inhibiting β cell apoptosis [25]. However, the mechanism of GLP-1 in regulating insulin secretion is still not clearly clarified.

We hypothesized that GLP-1RA improves the HOMA-β by regulating the level of Wnt5a protein. To investigate our hypothesis, we treated newly diagnosed type 2 diabetes patients with GLP-1RA for 3 months to explore the relationship between GLP-1RA, Wnt5a protein, and HOMA-β.

## Material and methods

### Patient recruitment and exclusion criteria

This study was carried out at the endocrinology department of Xuzhou Central Hospital from June 2020 to March 2021 in line with the principles enunciated in the Declaration of Helsinki. This study was reviewed and approved by the Ethical Committees of Xuzhou Central Hospital (Ethical approval NumberXZXY-LJ-20200902-035). Written informed consent was obtained from all of the participants.

56 patients with elevated blood glucose were selected from the Endocrinology Department of Xuzhou Central Hospital. The diagnosis of T2DM was based on the American Diabetes Association (ADA) 2014 criteria. Patients with onset T2DM had a plasma fasting glucose level > 7.0 mmol/L or 2 h values in the OGTT >11.1 mmol/L without a previous diabetes diagnosis. Subjects meeting any of the following criteria were excluded from this study: (1) T1DM; (2) acute complications of diabetes, such as diabetic ketoacidosis, hyperglycemic hyperosmolar state, lactic acidosis and hypoglycemic coma; (3) pregnancy; (4) Patients with heart failure, hematologic disease, liver dysfunction, or taking steroids were also excluded. After being divided into intervention group 29 cases (GLP-1RAs 1.2 mg subcutaneous injection once a day, liraglutide, Novo Nordisk), 27 cases in the control group (Metformin, 0.5 g twice a day, Glucophage, Merck). Individuals who were using medications to manage platelet (Aspirin) and cholesterol (Statins) were enrolled and continued treatment throughout the study (total 12 weeks).

### Clinical and biochemical evaluation

Research methods All subjects underwent a 75 g oral glucose tolerance test before and after treatment, and tested the following indicators on an empty stomach in the early morning: height (m), weight (kg), body mass index (BMI), waist-to-hip ratio (WHR), blood pressure, fasting blood glucose, fasting Insulin, HbA1C, TG, TC, HDL-C and LDL-C, Hcy levels, and calculate Homa-β = 20 × FINS/(FPG-3.5); follow up every 4 weeks for a total of 12 weeks; follow-up content: early morning Fasting weight, waist circumference, hip circumference, FPG and PPG, as well as liver and kidney function, and provide diabetes education to patients to understand the patient’s diet and exercise treatment; Measurement method Blood glucose and blood lipids (TG, TC, HDL-C and LDL-C) were measured by Hitachi 7600 automatic biochemical analyzer; HbA1C was measured by high pressure liquid method; Wnt5a and Sfrp5 concentrations were determined using ELISA kit (ISBio, United States of America), respectively. All specimens are tested in the same batch, and the intra-assay difference is <5%.

### Statistical methods

The means ± standard deviation were used to represent continuous data. Two independent samples t test was performed to analyze the continuous data before or after treatment. The changed data was analyzed by the paired t test. Multivariate linear regression was used to explored the association between △HOMAβ and △Wnt5a or △Sfrp5. SPSS for Windows version 22.0 (IBM Co., Armonk, NY, USA) was used for performing all statistical analyses in the study. Statistical significance was considered for P values less than 0.05.

## Results

### The clinical characteristics of participants before treatment

The Table 1 shows that there were no statistical differences of clinical characteristics between the control group and the intervention group.

**Table 1:** The comparison of clinical characteristics of participants before treatment

Variables	Control group	Intervention group	*P*
*n*	27	29	
Sex(male)	14	18	0.441
Age(years)	46.47 ± 3.57	47.23 ± 5.84	0.592
Weight(Kg)	78.89 ± 18.40	83.14 ± 21.63	0.434
BMI(Kg/m^2^)	27.15 ± 4.59	27.75 ± 5.89	0.676
Fins(mU/L)	13.19 ± 12.79	10.79 ± 10.28	0.449
FPG(mmol/L)	9.56 ± 3.27	10.28 ± 3.50	0.437
HbA1c(%)	9.17 ± 2.09	9.39 ± 2.54	0.723
WHR(%)	0.94 ± 0.05	0.96 ± 0.09	0.371
HOMAβ^#^	1.62 ± 0.47	1.45 ± 0.44	0.167
HOMAIR^#^	0.61 ± 0.31	0.54 ± 0.33	0.485
TC(mmol/L)	4.97 ± 0.81	4.92 ± 1.08	0.841
HDL-C(mmol/L)	0.96 ± 0.22	1.10 ± 0.26	0.063
Wnt5a (ng/ml)	54.04 ± 5.23	55.24 ± 5.79	0.421
Sfrp5 (ng/ml)	43.37 ± 8.22	45.35 ± 8.81	0.388

#: The variables were transferred by ln; *BMI* body mass index, *Fins* fasting insulin, *HbA1c* Glycosylated Hemoglobin, *WHR* waist hip rate, *HOMA-β* homeostasis model assessment of β-cell function, *HOMA-IR* homeostasis model assessment of insulin resistance, *TC* total cholesterol, *HDL-C* high-density lipoproteincholesterol

### During of treatment compared the changed value of clinical characteristics between intervention group and control group

The Table 2 shows that the change value of clinical characteristics between two groups have statistical difference. The participants with the intervention group have higher changed value than the control group.

**Table 2:** The comparison of changed value of clinical characteristics of two groups during of treatment

Variables	Control group	Intervention group	*P*
	Changed value	Changed value	
*n*	27	29	
Weight(Kg)	1.17 ± 0.27	5.05 ± 0.56	<0.001
BMI(Kg/m2)	0.41 ± 0.11	1.71 ± 0.26	<0.001
Fins(mU/L)	1.91 ± 0.75	3.82 ± 1.04	<0.001
FPG(mmol/L)	1.52 ± 0.57	3.57 ± 0.76	<0.001
HbA1c(%)	1.29 ± 0.45	2.74 ± 0.86	<0.001
WHR(%)	0.09 ± 0.001	0.30 ± 0.01	<0.001
HOMAβ^#^	23.80(9.91 ~ 69.03)	77.55(17.88 ~ 140.07)	0.001
HOMAIR^#^	0.05(−0.46 ~ 0.44)	−0.49(−1.20 ~ 0.85)	<0.001
TC(mmol/L)	1.00 ± 0.37	2.14 ± 0.12	<0.001
HDL-c(mmol/L)	0.42 ± 0.03	0.50 ± 0.04	<0.001
Wnt5a(ng/ml)	2.87 ± 1.39	8.97 ± 1.76	<0.001
Sfrp5(ng/ml)	−3.28 ± 1.83	−10.48 ± 2.34	<0.001

*BMI* body mass index, *Fins* fasting insulin, *HbA1c* Glycosylated Hemoglobin, *WHR* waist hip rate, *HOMA-β* homeostasis model assessment of β-cell function, *HOMA-IR* homeostasis model assessment of insulin resistance, *TC* total cholesterol, *HDL-C* high-density lipoproteincholesterol

### The comparison of the clinical characteristics after treatment between two groups

After treatment, the clinical characteristics of the intervention group compared with those of the control group have statistical difference. WHR and Sfrp 5 of the intervention group were lower than those of the control group; HOMAβ, TC, and Wnt5a of the intervention group were higher than those of the control group (Tables 3 and 4).

**Table 3:** The comparison of the clinical characteristics after treatment between two groups

	Control group	Intervention group	*P*
*n*	27	29	
Weight(Kg)	77.72 ± 18.45	78.09 ± 21.67	0.946
BMI(Kg/m2)	26.74 ± 4.63	26.18 ± 5.77	0.691
Fins(mU/L)	15.29 ± 12.65	14.62 ± 10.32	0.829
FPG(mmol/L)	8.30 ± 3.00	7.18 ± 2.96	0.166
HbA1c(%)	7.88 ± 2.16	6.79 ± 2.42	0.081
WHR(%)	0.84 ± 0.06	0.66 ± 0.09	<0.001
HOMAβ#	1.87 ± 0.52	1.96 ± 0.50	<0.001
HOMAIR#	0.63 ± 0.28	0.58 ± 0.23	0.421
TC(mmol/L)	5.97 ± 0.95	7.05 ± 1.03	<0.001
HDL-c(mmol/L)	1.39 ± 0.22	1.39 ± 0.22	0.918
Wnt5a(ng/ml)	56.91 ± 5.32	64.22 ± 6.36	<0.001
Sfrp5(ng/ml)	40.09 ± 8.74	34.87 ± 9.46	0.037

*BMI* body mass index, *Fins* fasting insulin, *HbA1c* Glycosylated Hemoglobin, *WHR* waist hip rate, *HOMA-β* homeostasis model assessment of β-cell function, *HOMA-IR* homeostasis model assessment of insulin resistance, *TC* total cholesterol, *HDL-C* high-density lipoproteincholesterol

**Table 4:** The association between △HOMAβ and △Wnt5a or △Sfrp5

	Beta	S.E	t	*P*
△Wnt5a	41.12	9.371	4.385	<0.001
△Sfrp5	−24.33	7.122	−3.416	0.001

### The association between △HOMAβ and △Wnt5a or △Sfrp5

Using multivariate linear regression, we found there were a positive correlation between △HOMAβ and △Wnt5a level, and a negative correlation between △HOMAβ and △Sfrp5 levels.

### The association between △HOMAβ and △Wnt5a or △Sfrp5 in different groups

**Fig. 1:**
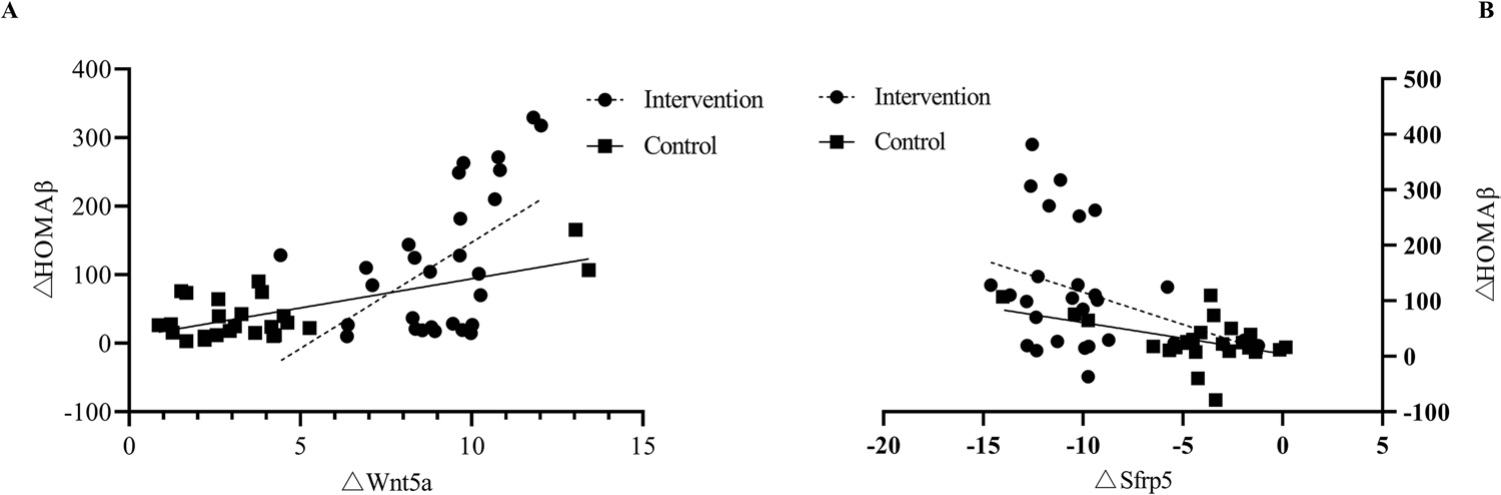
The association between △HOMAβ and △Wnt5a or △Sfrp5 in different groups. **A** There is a positive relationship between △HOMAβ and △Wnt5a in two groups, and the slope of linear regression of the positive relationship in intervention is higher than those in control group, the P value is <0.001. **B**: There is a negative relationship between △HOMAβ and △Sfrp5 in two groups, and the intervention group had a higher slope curve than the control groups

The △HOMAβ of all groups were increased with △Wnt5a levels, and the intervention group had a higher slope curve than the control groups (Fig. 1A). The △HOMAβ of all groups were reduced with △Sfrp5 levels, and the intervention group had a higher slope curve than the control groups (Fig. 1B). The Fig. 1 shows the changed tread of HOMAβ was more significance in the intervention group.

## Discussion

The current study found that glucagon-like peptide 1 receptor agonists can improve the islet secretion index of newly diagnosed type 2 diabetes patients. More interestingly, the serum Wnt5a protein level increased significantly after the intervention of the GLP-1RA, and the Sfrp5 decreased compared with the control group. The change of Wnt5a protein level is positively correlated with the change of HOMA-β. Previous studies have confirmed that Wnt5a protein is involved in the regulation of insulin secretion in pancreatic beta cells. Based on our understanding, this is the first time to explore the effect of GLP-1RA on the level of Wnt5a protein.

It is believed that insulin resistance and/or insufficient secretion are the main pathogenesis of diabetes, and pancreatic islet dysfunction plays an important role in the occurrence and development of diabetes [26]. With the deepening of research, glucose toxicity, lipotoxicity, oxidative stress, mitochondrial stress, low-level inflammation, and brain-gut-pancreatic axis dysfunction are all involved in the occurrence of pancreatic islet dysfunction. The evaluation of islet function plays an important role in the identification of diabetes types and the guidance of clinical medication. The methods currently used for the evaluation of islet function include the positive glucose clamp test, the miniature model method, the area under the insulin curve after glucose load, and the arginine test [8–12]. However, due to the complexity of the above detection methods, they cannot be fully promoted in clinical practice. At present, fasting insulin and blood glucose levels are usually used to calculate HOMA-β to estimate the insulin secretion capacity of patients in clinical practice. Although the results are easily affected by insulin resistance factors, it is widely used in clinical practice because of its simplicity [27]. Our study found that newly diagnosed type 2 diabetes patients received GLP-1RA treatment, their insulin secretion index increased significantly, and the insulin resistance index decreased compared with the control group. It has been confirmed once again that GLP-1RA can improve the islet secretion of type 2 diabetic patients and can reduce insulin resistance to play an important role in the treatment of diabetes.

Wnt5a is one of the important members of the non-classical Wnt family, which participates in many physiological and pathological processes such as tissue fibrosis and tumorigenesis. With the in-depth understanding of the relationship between adipose tissue and the endocrine system, studies have confirmed that adipose inflammatory factors such as adiponectin, tumor necrosis factor, and interleukin are involved in the occurrence and development of diabetes through their own mechanisms [28]. As one of the important cytokines secreted by adipose tissue, the research on Wnt5a and diabetes has attracted the attention of the majority of researchers. Previous studies have found that Wnt5a protein and its receptor Fzd5 are involved in the process of pancreatic tissue formation and islet development [29]. As an antagonist of Wnt5a protein, SFRP5 protein can competitively bind to wnt5a protein [30]. Clinical studies have found that the level of SFRP5 protein in patients with type 2 diabetes is significantly higher than that of normal people [31]. Our previous studies also confirmed that the level of Wnt5a in patients with type 2 diabetes was significantly reduced in patients with newly-onset type 2 diabetes [18], and that Metformin treatment can increase the level of serum Wnt5a protein in newly diagnosed patients with type 2 diabetes. The paracrine Wnt5a of stellate cells in the pancreatic islets regulates the secretion of insulin in beta cells through the non-classical Wnt/Ca pathway and Fox01 signaling pathway [19, 20]. Our study found that the serum Wnt5a protein level of newly diagnosed type 2 diabetes patients was significantly increased after the intervention of the glucagon-like peptide-1 receptor agonist compared with before treatment, and the antagonist Sfrp5 was lower than that of the control group. The increase of wnt5a protein level after GLP-1RA intervention treatment was higher than that in the metformin treatment group. The change of Wnt5a protein level is positively correlated with the change of HOMA-β. Our results confirm that Wnt5a protein is closely related to pancreatic islet dysfunction in type 2 diabetes, and it may be a key protein for GLP-1RA to promote insulin secretion in beta cells.

GLP-1 (glucagon-like peptide-1), a brain-gut peptide secreted by ileal endocrine cells, can inhibit gastric emptying and reduce bowel movement, so it helps control food intake and reduce weight. Studies have found that glucagon-like peptide-1 can promote beta cell proliferation through the classic Wnt/β-cat/TCF7L2 pathway [32]. Liraglutide suppresses production of extracellular matrix proteins and ameliorates renal injury of diabetic nephropathy by enhancing Wnt/β-catenin signaling [33]. Interestingly, Exendin-4 promotes pancreatic β-cell proliferation via inhibiting the expression of Wnt5a [33]. Our study found that the serum Wnt5a protein level of newly diagnosed type 2 diabetes patients was significantly increased after the intervention of the GLP-1RA compared with before treatment, and the Sfrp5 was lower than that of the control group. Our results confirm that GLP-1RA can not only interact with beta cells through the classical Wnt signaling pathway, but also through the non-classical Wnt5a protein. These results add new explanations to the mechanism by which GLP-1RA improve insulin secretion. Wnt5a protein may play different roles in different stages of diabetes. Our previous studies confirmed that its level in the serum of newly diagnosed type 2 diabetes patients was significantly lower than that in healthy controls, but it is interesting that the serum level of diabetic nephropathy patients increased significantly. The possible explanation is that the physiological concentration of Wnt5a protein is involved in the regulation of insulin secretion in beta cells, and it participates in the process of tissue and organ repair and even fibrosis as feedback increases in the level of inflammatory factors in the stage of diabetic complications. The sample size of our research is small, and there is a lack of basic research mechanisms. We are conducting animal and cell experiments on the effect of GLP-1RA on wnt5a protein, hoping to provide a better mechanism explanation for the clinical phenomenon.

In summary, we found that the Wnt5a protein are involved in the pathological process of type 2 diabetes. GLP-1RA increases Wnt5a and affects the insulin secretion index. These data presented here may provide new insights into the mechanism of GLP-1RA in the treatment of type 2 diabetes patients and specific targeting Wnt5a may be a potential therapy.

## Data Availability

Please contact the corresponding author with data requests.
